# Plasma interleukin-37 is increased and inhibits the production of inflammatory cytokines in peripheral blood mononuclear cells in systemic juvenile idiopathic arthritis patients

**DOI:** 10.1186/s12967-018-1655-8

**Published:** 2018-10-11

**Authors:** Miao Feng, Min Kang, Feng He, Zonghui Xiao, Zhewei Liu, Hailan Yao, Jianxin Wu

**Affiliations:** 1 Department of Biochemistry and Immunology, Capital Institute of Pediatrics, No. 2 Yabao Road, Chao Yang District, Beijing, 100020 China; 2 Department of Immunology and Rheumatology, Capital Institute of Pediatrics, No. 2 Yabao Road, Chao Yang District, Beijing, 100020 China

**Keywords:** Interleukin-37, Systemic juvenile idiopathic arthritis, Inflammatory cytokines, Peripheral blood mononuclear cells

## Abstract

**Background:**

Interleukin (IL)-37 has emerged as a novel anti-inflammatory cytokine that play an immunosuppressive role in regulating inflammatory response. This study aimed to measure IL-37 levels in the plasma and peripheral blood mononuclear cells (PBMCs) of patients with systemic juvenile idiopathic arthritis (sJIA), and to establish the correlation between IL-37 levels and disease activity, laboratory parameters and inflammatory cytokines.

**Methods:**

The mRNA levels of IL-37 in PBMCs and plasma IL-37 concentrations in 46 sJIA patients and 30 age- and sex-matched healthy controls were measured by real-time polymerase chain reaction (RT-PCR) and enzyme-linked immunosorbent assay (ELISA), respectively. The correlations between plasma IL-37 levels and disease activity, laboratory parameters and inflammatory cytokines in sJIA were analyzed by Spearman correlation test. PBMCs from the sJIA patients were stimulated with recombinant human IL-37 (rhIL-37) protein, expressions of IL-1β, IL-6, TNF-α and IL-17 were detected by RT-PCR and ELISA.

**Results:**

Plasma levels of IL-37 and relative IL-37 mRNA expression were significantly elevated in sJIA patients, especially in active sJIA patients, when compared with the healthy controls (*P* < 0.001). Furthermore, patients with active disease showed higher IL-37 mRNAs and plasma protein levels than those with inactive disease as well as healthy controls. Plasma IL-37 levels were correlated with disease activity and inflammatory cytokines (IL-6, TNF-α, IL-17 and GM-CSF) in sJIA patients. The productions of inflammatory cytokines such as IL-6, TNF-α, IL-17 in PBMCs from sJIA patients were obviously decreased after recombinant IL-37 stimulation, whereas the production of IL-1β was not changed.

**Conclusions:**

Our results demonstrate that levels of IL-37 were higher in sJIA patients, which were correlated with disease activity and sJIA related inflammatory cytokines. In addition, rhIL-37 down-regulates the expressions of inflammatory cytokines form PBMCs in sJIA patients, suggesting that IL-37 may have the potential role as a natural inhibitor for the pathogenesis and therapy of sJIA.

**Electronic supplementary material:**

The online version of this article (10.1186/s12967-018-1655-8) contains supplementary material, which is available to authorized users.

## Background

Systemic juvenile idiopathic arthritis (sJIA) is a seriously rheumatic and chronic auto-inflammatory disease characterized by the dysregulated systemic inflammation (hepatosplenomegaly, serositis, lymphadenopathy, hectic quotidian fevers, typical rash, acute-phase reaction), which seriously threatens the life of children [1]. Although the pathogenesis of sJIA remains unclear, more and more research indicated that inflammatory cytokines were involved in the regulation of systemic inflammation, local joints damage and bone erosion [2]. It seems that the balance between pro- and anti-inflammatory cytokines may play a vital role in the pathogenesis and progression of sJIA.

Abundant evidences have suggested that pro-inflammatory cytokines, such as interleukin (IL)-6, IL-17 and tumor necrosis factor-α (TNF-α), were significantly increased in the peripheral blood of sJIA patients, which are related to the pathogenesis of sJIA [3–5]. Interestingly, although IL-1β serum levels in sJIA patients were as low as those of healthy controls (HCs), neutralization of IL-1β with anti-IL-1β agents could effectively and sustainably block the inflammatory response in sJIA [6, 7]. Accumulating clinical trials demonstrated that therapies targeting these cytokines or their receptors could partially alleviate the inflammatory symptoms and reduce disease activity in sJIA [8–10]. However, the studies related to the expression and function of anti-inflammatory cytokines in sJIA remain poorly understood. IL-37, formerly named IL-1F7, downregulated the expression of pro-inflammatory cytokines in various inflammatory diseases [11], such as ankylosing spondylitis (AS), systemic lupus erythematosus (SLE), rheumatoid arthritis (RA), and adult-onset Still’s disease (AOSD) [12–16], then relieving the inflammatory responses in sJIA.

IL-37, as a recently identified member of IL-1 family, has been shown an important anti-inflammatory cytokine in the development of many inflammatory diseases, autoimmune diseases and tumors [11, 17]. IL-37 is widely expressed in human tissues such as thymus, lymph nodes, testis and bone marrow, and in various types of cells such as peripheral blood monocytes (PBMCs), epithelial cells and dendritic cells [18]. The IL-37 expression level is low and IL-37 mRNA is easily degraded in healthy human tissues and cells [17–19]. When it is stimulated by lipopolysaccharide (LPS) or pro-inflammatory cytokines, the expression level of IL-37 is significantly upregulated and stability of IL-37 mRNA is markedly enhanced, suggesting IL-37 might play a primary role in suppressing excessive immune response [17]. In recent years, accumulating studies have found that IL-37 is closely related to inflammatory and autoimmune diseases, including SLE, RA, and AOSD [13–16, 20–22]. Concomitantly, IL-37 can inhibit the production of pro-inflammatory cytokines in PBMCs from subjects with SLE, RA, AS and AOSD in vitro. As yet, only one study investigated IL-37 levels in the serum and synovial fluid of JIA patients and demonstrated the positive correlation with disease activity and markers of angiogenesis [23]. Although the IL-37 levels and IL-37 mRNA expression have been reported to be elevated in JIA patients, especially in sJIA patients, when compared with the healthy subjects, its relationships with other disease manifestations and pro-inflammatory cytokines are still unknown. Moreover, whether IL-37 can inhibit the expression of inflammatory cytokines in PBMCs from sJIA patients to protect the development of sJIA remains to be explored.

Here, we measured the expressions of IL-37 mRNA in PBMCs and IL-37 protein in plasma of patients with sJIA and healthy controls, and analyzed the correlation of plasma IL-37 levels with disease activity, laboratory parameters and inflammatory cytokines in sJIA. In addition, we assessed the effect of IL-37 on cytokine production in PBMCs from patients with sJIA.

## Methods

### Patients and healthy control subjects

Forty-six sJIA patients diagnosed according to the International League Against Rheumatism classification were recruited from the Department of Rheumatology and Immunology at the Capital Institute of Pediatrics Hospital from 2016 to 2018 [24]. Thirty age- and sex-matched healthy subjects with no history of any rheumatic, autoimmune disease were involved as healthy controls (HCs). Our study was approved by the ethics committee of the Capital Institute of Pediatrics. Written informed consent was obtained from each of the participants and their guardians.

Plasma samples of the patients with active sJIA were enrolled before any kind of nonsteroidal anti-inflammatory drug, disease-modifying anti-rheumatic drug, glucocorticoid, immune-suppressor, or biological agent treatments. All subjects were subjected to the medical histories and clinical characteristics. Clinical data from each patients were recorded (Table 1). Disease activity was measured using the Juvenile Arthritis Disease Activity Score in 27 joints (JADAS-27), which included 4 measures: physician global assessment of disease activity, parent/patient global assessment of well-being, active joint count, and erythrocyte sedimentation rate (ESR) [25]. The JADAS-27 was calculated as the simple linear sum of the scores of its 4 components, which yields a global score of 0 (no activity)—57 (high activity).

**Table 1:** Clinical characteristics of sJIA patients and healthy controls

Characteristics	Active sJIA (n = 23)	Inactiv sJIA (n = 23)	sJIA (n = 46)	HCs (n = 30)
Age (years)	7.3 ± 3.1	7.4 ± 3.3	7.4 ± 3.2	6.6 ± 2.8
Sex, no. F/M	13/10	7/16	20/26	12/18
Disease duration (years)	2.2 ± 0.88	2.0 ± 0.83	2.1 ± 0.86	N/A
JADAS-27	26.09 ± 7.48	13.94 ± 6.82	20.02 ± 9.37	N/A
ESR, mm/h	48.69 ± 5.64	35.77 ± 6.89	41.53 ± 9.69	N/A
CRP, mg/L	25.87 ± 6.17	21.47 ± 3.36	23.7 ± 4.43	N/A
PLT, 10^9^/L	361.39 ± 91.94	350.22 ± 90.98	357.98 ± 90.37	N/A
ALT, U/L	20.07 ± 19.16	24.73 ± 20.72	22.41 ± 19.87	N/A
AST, U/L	33.32 ± 20.00	36.20 ± 18.97	34.76 ± 19.53	N/A
ANA positivity (%)	3 (13.04)	0 (0)	3 (6.52)	N/A
RF positivity (%)	0 (0)	0 (0)	0 (0)	N/A

For age, disease duration, JADAS-27, ESR, CRP, PLT, ALT and AST, data are presented as median

N/A, not available

### Blood collection and PBMCs isolation

Venous blood samples were collected within four hours and directly transferred into ethylene diamine tetraacetic acid (EDTA)-treated tubes. After centrifuged at 2000*g* for 4 min at room temperature, aliquots of the supernatant were transferred into new RNase-free tubes and stored at − 80 °C until cytokines were determined. PBMCs were isolated from sJIA patients and HCs using Lymphocyte Separation Medium (MP Biomedicals, USA) under sterile conditions for cell culture or frozen at − 80 °C untile RNA extraction.

### Expression and purification of recombinant human IL-37 (rhIL-37) protein

Human IL-37 gene, amplified from cDNA of PBMCs using the primer pair 5′-CGGGATCCATGGTTCACACAAGTCCA-3′ and 5′-CCCAAGCTTCTAATCGCTGACCTCACT-3′, were cloned into pET21a vector and expressed in *E. coli* BL21 (DE3) cells. Protein expression was induced by 0.4 mM isopropyl β-D-thiogalactopyranoside in lysogeny broth (LB) medium and cells were cultured for an additional 6 h at 37 °C. Cells were then harvested by centrifugation and resuspended in lysis buffer (NaCl–Tris–HCl), sonicated in an ice bath, centrifuged at 20,000*g* for 30 min. The soluble fraction was loaded to His Trap HP, 1 ml column (GE) pre-equilibrated with lysis buffer and the proteins were eluted with different concentrations of imidazole buffer. Target protein was examined by SDS-PAGE electrophoresis and dialyzed in PBS at 4 °C for overnight. The concentrations were detected by Brandford methods, and the recombinant protein was stored at − 80 °C.

### Cell culture and rhIL-37 treatment

PBMCs were cultured in RPMI 1640 (Gibco, Thermo Fisher Scientific, USA) with 10% fetal calf serum (Gibco, Thermo Fisher Scientific, USA), 100 μg/ml streptomycin (Beyotime, China) and 100 IU/ml penicillin, and in a humidified atmosphere of 5% CO_2_ at 37 °C. Cells were cultured at 1.5 × 10^6^ cells/ml in 48-well plates in the absence or presence of rhIL-37 at various concentrations. After 6 h, one group of the cells were incubated further with 1 μg/ml LPS (Sigma-Aldrich, USA) for 6 h, and then total RNAs were extracted and cytokine transcriptions were analyzed by RT-PCR. Another group of the cells were incubated further with 1 μg/ml LPS after 24 h. 6 h later, culture supernatants were harvested and frozen at − 80 °C for cytokine analysis by ELISA.

### RNA extraction and RT-PCR

RNA samples were extracted from PBMCs by Trizol regent (Invitrogen, USA), according to the manufacturer’s instructions. cDNAs were obtained using the RT System A3500 Kit (Promega, USA). The primer sequences were summarized in Table 2. RT-PCR amplification reactions were performed using the SYBR Green Real-Time PCR assay and operated by the QuantStudio 6 Flex Real-Time PCR System (Applied Biosystems). PCR products were amplified in duplicate in a total volume of 20 μl, verified by melting curve analysis. Relative mRNAs levels of target genes were calculated with normalization to β-actin values using the 2^−ΔΔct^ method.

**Table 2:** List of the sequence of human gene primers

Gene name	Forward (5′–3′)	Reverse (5′–3′)
IL-37	AGTGCTGCTTAGAAGACCCGG	AGAGTCCAGGACCAGTACTTTGTGA
TNF-α	ACCTCTCTCTAATCAGCCCTCT	GGGTTTGCTACAACATGGGCTA
IL-1β	CCACAGACCTTCCAGGAGAAT	GTGCACATAAGCCTCGTTATCC
IL-6	ACTCACCTCTTCAGAACGAATTG	CCATCTTTGGAAGGTTCAGGTTG
IL-17	ACCTCATTGGTGTCACTGCTAC	GTTCAGGTTGACCATCACAGTC
β-actin	CCTGACTGACTACCTCATGAAG	GACGTAGCACAGCTTCTCCTTA

### Cytokine assessment

IL-37 protein levels were quantified using ELISA reagent kits purchased from AdipoGen Life Sciences (San Diego, CA, USA). The following cytokines IL-1β, IL-6, TNF-α, IFN-γ and GM-CSF in plasma were measured using the Milliplex MAP Human High Sensitivity T Cell Panel Premixed 13-plex kit (Cat. No. HSTCMAG28SPMX13, EMD Millipore, Billerica, MA 01821, USA), and then detected using Luminex Xmap multiplex technology. Plasma IL-17 levels and cell culture supernatants IL-1β, IL-6, TNF-α, and IL-17 levels were determined using the Proteintech ELISA kits (San Diego, CA, USA).

### Statistical analysis

Statistical analyses were analyzed using Graphpad Prism V.5.00 software (GraphPad Software, San Diego CA, USA). All descriptive variables were expressed as mean (±SEM) or median (range). Comparisons between 2 groups were performed by the Student’s *t* test or Mann–Whitney U-test for nonparametric data. Spearman correlation test was used to evaluate the associations between plasma IL-37 levels and different variables. The P values < 0.05 were considered statistically significant.

## Results

### Increased expression of IL-37 mRNA and plasma protein levels in patients with sJIA

**Fig. 1:**
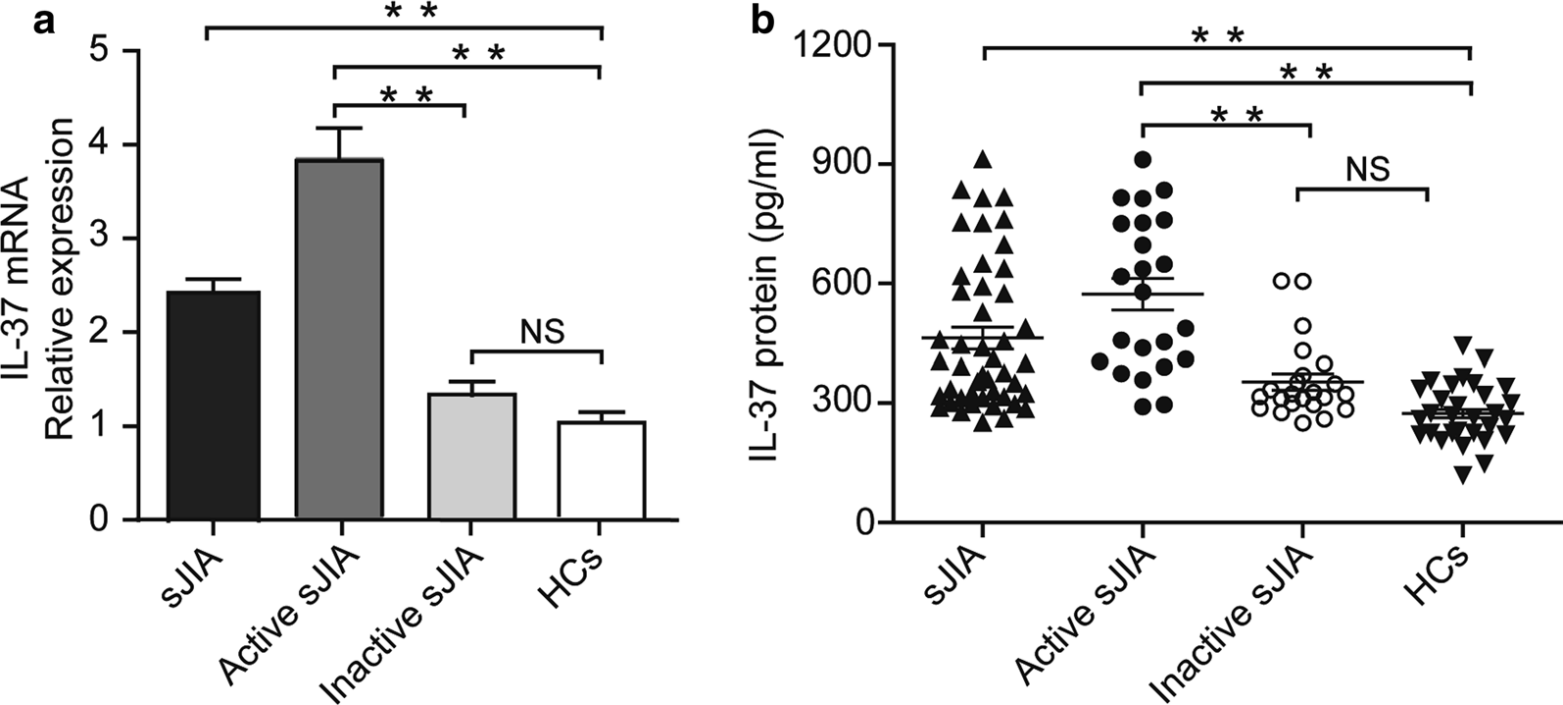
Comparison of IL-37 levels between sJIA and HCs. **a** Expression of IL-37 mRNA in PBMCs from sJIA patients (n = 46), distributed according to disease activity (active, n = 23; inactive, n = 23), and HCs (n = 30) was determined by real-time PCR. **b** Plasma IL-37 levels in sJIA patients (n = 46), distributed according to disease activity (active, n = 23; inactive, n = 23), and HCs (n = 30) were measured by ELISA. Horizontal lines indicate median values. Differences between two groups were performed with Mann–Whitney U-test for nonparametric data. sJIA, systemic juvenile idiopathic arthritis; HCs, healthy controls; NS, not significant. **P < 0.001 by Student’s *t* test

To investigate the potential role of IL-37 in patients with sJIA, 46 sJIA patients and 30 age- and sex- matched HCs were enrolled. IL-37 mRNA expression in PBMCs was measured by RT-PCR and the plasma protein levels were detected by ELISA. The results showed that IL-37 mRNA and plasma protein levels were significantly higher in sJIA patients compared with HCs (Fig. 1), indicating that IL-37 probably participated in the pathogenesis of sJIA. Next, we divided sJIA patients into active (n = 23) and inactive (n = 23) groups, according to the JADAS-27 scores. We found a dramatically upregulation of IL-37 mRNA and plasma protein levels in active sJIA patients compared with inactive sJIA patients (Fig. 1). Besides, no difference was detected in IL-37 mRNA and plasma IL-37 protein levels between inactive sJIA patients and HCs (Fig. 1).

### Correlation between IL-37 levels and sJIA disease activity as well as laboratory indexes

**Fig. 2:**
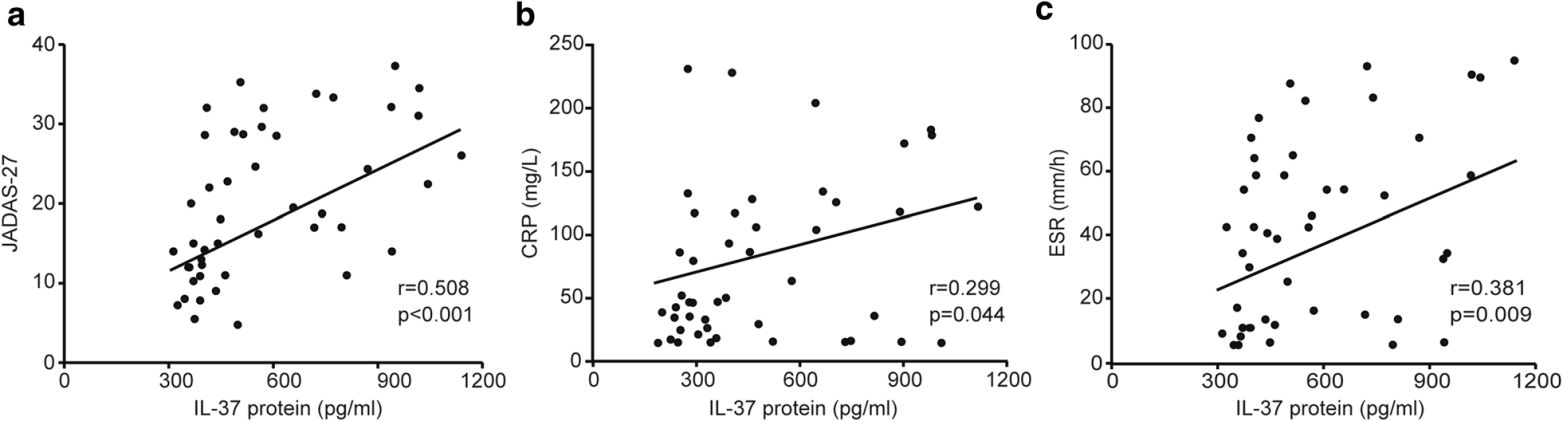
Correlations of plasma IL-37 levels and laboratory values in patients with sJIA. Plasma IL-37 levels were positively correlated with JADAS-27 (**a**), CRP (**b**), and ESR (**c**) respectively. Each symbol represents an individual patient. The correlations were evaluated with Spearman’s nonparametric test. P < 0.05 represents a significant difference. JADAS-27, Juvenile Arthritis Disease Activity Score in 27 joints. ESR, erythrocyte sedimentation rate; CRP, C-reactive protein

Next, we examined the potential relationship of IL-37 levels with disease activity, defined by JADAS-27, as well as laboratory values, including C-reactive protein (CRP), erythrocyte sedimentation rate (ESR), platelet (PLT), alanine transaminase (ALT) and aspartate transaminase (AST). The results revealed that a significantly positive correlation was observed between plasma IL-37 levels and JADAS-27 (r = 0.508, *P* < 0.001) (Fig. 2a). Similarly, there was a positive correlation between plasma IL-37 levels and CRP (r = 0.299, *P* = 0.044) and ESR (r = 0.381, *P* = 0.009), respectively (Fig. 2b, c). No significant correlation was found between plasma levels of IL-37 and PLT (r = 0.113, *P* = 0.453), ALT (r = -0.106, *P* = 0.482) and AST (r = 0.02, *P* = 0.894).

### Associations of plasma IL-37 levels with inflammatory cytokines levels in sJIA

**Fig. 3:**
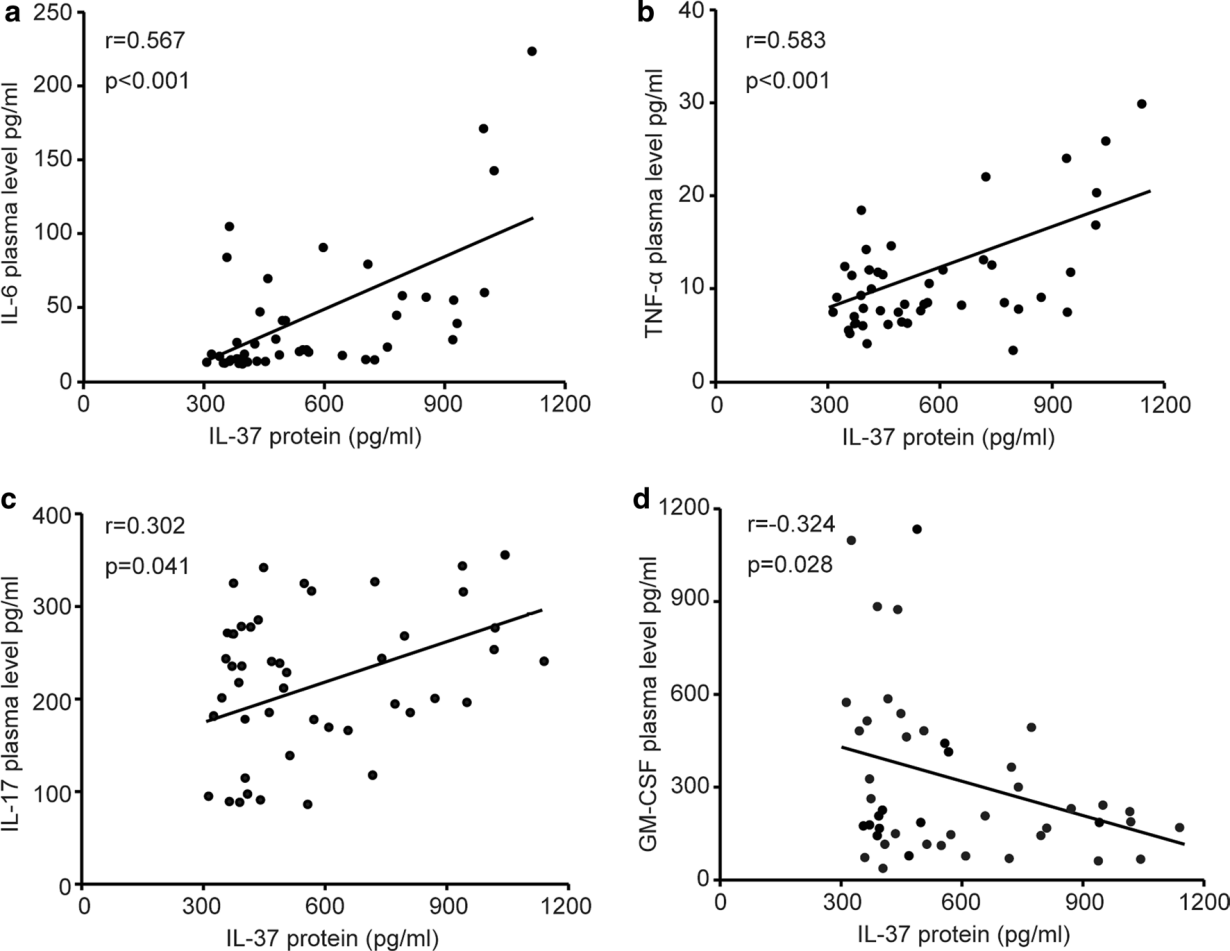
Correlations of plasma IL-37 levels and inflammatory cytokines in patients with sJIA. Plasma IL-37 levels were positively correlated with IL-6 (**a**), TNF-α (**b**), IL-17 (**c**) respectively. Plasma IL-37 levels were negatively correlated with GM-CSF (**d**). Each symbol represents an individual patient. The correlations were evaluated with Spearman’s non-parametric test. TNF-α, tumor necrosis factor-α; GM-CSF, granulocyte–macrophage colony stimulating factor

Studies have shown that IL-37 can regulate the production of various pro-inflammatory cytokines, such as IL-1β, IL-6, TNF-α, IL-17, interferon-γ (IFN-γ) and granulocyte–macrophage colony stimulating factor (GM-CSF), and some of the cytokines have been reported to contributed to the multisystem inflammation of sJIA [18, 26]. To further survey whether plasma IL-37 levels were correlated with these pro-inflammatory cytokines, we first measured the plasma levels of these cytokines in patients with sJIA (Additional file 1: Fig. S1). In accordance with published research, the levels of plasma IL-6, TNF-α and IL-17 were significantly higher in active sJIA patients than in inactive sJIA patiens and in HCs. No significant differences were viewed in plasma IL-1β and IFN-γ between active and inactive sJIA patients. In addition, the inactive sJIA patients displayed higher plasma IL-1β, IL-6, TNF-α and IL-17 than those in HCs. Next, we carried out Spearman’s correlation method to assessed whether plasma IL-37 levels correlated with these cytokines. As seen in Fig. 3a–c, significantly positive correlations were observed between plasma IL-37 concentrations and the levels of plasma IL-6 (r = 0.576, *P* < 0.001), TNF-α (r = 0.583, *P* < 0.001) and IL-17 (r = 0.302, *P* = 0.041). No significant correlations were found between plasma IL-37 levels and IL-1β (r = 0.149, *P* = 0.321) and INF-γ (r = 0.217, *P* = 0.147). Besides, we also detect the levels of another cytokine GM-CSF in sJIA patients. The plasma GM-CSF levels were also lower in sJIA patients compared to HCs. Interestingly, there was a negative correlation between IL-37 and GM-CSF.

### IL-37 suppresses inflammatory cytokine expression in PBMCs from patients with sJIA

**Fig. 4:**
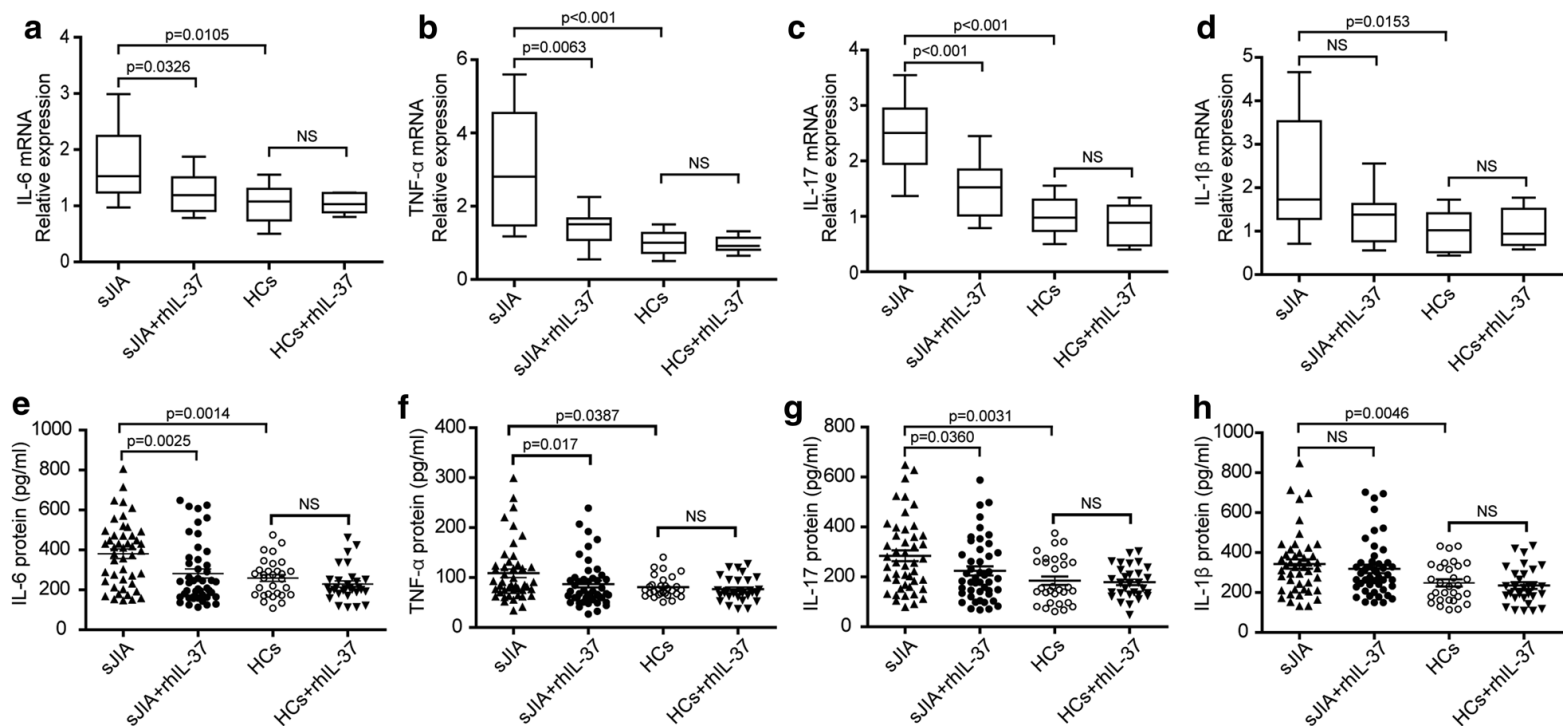
IL-37 inhibited the expression of inflammatory cytokines in PBMCs from patients with sJIA. PBMCs from sJIA patients (n = 46) and HCs (n = 30) were stimulated or not with rhIL-37 (100 ng/ml) for 6 h, cells then cultured with LPS (1 μg/ml) for 4 h, the mRNA levels of IL-6 (**a**), TNF-α (**b**), IL-17 (**c**) and IL-1β (**d**) were detected by RT-PCR. PBMCs from sJIA patients (n = 46) and HCs (n = 30) were stimulated or not with rhIL-37 (100 ng/ml) for 24 h and then incubated with LPS (1 μg/ml) for 6 h, and IL-6 (**e**), TNF-α (**f**), IL-17 (**g**) and IL-1β (**h**) levels in supernatant were assessed by ELISA. The data are expressed as mean ± SEM. P values are shown in the graph. NS, no significant. PBMCs, peripheral blood mononuclear cells; TNF-α, Tumor necrosis factor-α

To investigate whether IL-37 suppresses pro-inflammatory cytokines production in sJIA in vitro, we expressed and purified recombinant human IL-37 protein (rhIL-37). The PBMCs were isolated from sJIA patients and HCs, then cultured in the presence or absence of rhIL-37 and further with LPS stimulation. Through a dose-dependent manner, we found the optimum concentration of rhIL-37 and LPS was 100 ng/ml and 1 μg/ml, respectively. PBMCs from 46 sJIA patients and 30 HCs were either untreated or treated with rhIL-37 (100 ng/ml) for 6 or 24 h, and then treated with LPS for 6 h. The cells and cultural supernatants were harvested for real-time PCR and ELISA analysis, respectively. We found that IL-6, TNF-α and IL-17 in PBMCs of sJIA patients were obviously reduced by rhIL-37 (Fig. 4a–c). rhIL-37 also markedly suppress the secretion of IL-6, TNF-α and IL-17 in PBMCs of sJIA patients (Fig. 4e–g). However, the IL-1β cytokine mRNA (Fig. 4d) and protein levels (Fig. 4h) in PBMCs of sJIA were not noticeably changed by rhIL-37 treatment.

## Discussion

sJIA is one of the most perplexing rheumatic and chronic disease in children, presenting distinct disease symptoms compared to other subtypes of JIA [27]. The interesting feature of sJIA is the manifestation of systemic inflammatory in combination with chronic arthritis. The pathogenesis of its inflammatory response is related to a number of regulatory events, ranging from excessive activation of the immune system and inappropriately high levels of pro-inflammatory cytokines [1, 3, 28]. However, current research into the functions of anti-inflammatory cytokines in sJIA remains limited. IL-37 has been found as a natural inhibitor of inflammation and plays an important regulatory role in immune response by suppressing excessive inflammation [18, 26]. Studies have confirmed that IL-37 can attenuate inflammation in chronic inflammatory diseases, such as SLE, RA, AS and ASOD [12, 13, 16, 29]. Based on the above findings, we are eager to know whether and how IL-37 regulates inflammation induced by inflammatory cytokines in sJIA.

Recent data indicated that IL-37 expression was significantly elevated in plasma, PBMCs and synovial fluid (SF) in JIA patients [23]. Consistent with the findings, our results also revealed that plasma IL-37 levels were higher in sJIA patients compared with HCs. Moreover, we further investigated that plasma IL-37 levels were significantly elevated in patients with active disease than in patients with inactive disease and in HCs. The results suggest that IL-37 expression level is closely related to the internal inflammatory state. We also found that the mRNA level of IL-37 in PBMCs from patients with active sJIA was much higher than that from those with inactive sJIA and HCs, suggesting that an elevated level of inflammation may promote the expression of IL-37 mRNA in PBMCs to alleviate inflammatory response. In view of the constitutively low expression of IL-37 in non-inflammatory or mild inflammatory state, we speculated that IL-37 may primarily restrain excessive inflammation under severe inflammatory conditions.

The monitoring of sJIA disease activity is traditionally based on clinical observations and laboratory features, such as JADAS-27, CRP, ESR, PLT and so on [23, 30, 31]. We further analyzed the relationship between IL-37 expression and several laboratory values. We observed the positively correlation between the levels of IL-37 and several useful markers of the disease activity, including JADAS-27, CRP and ESR, but it lacked association with other laboratory values, including PLT, AST and ALT. As we known, JADAS is a valid instrument for the assessment of disease activity in JIA, and CRP concentrations and ESR values are also reliable biochemical indicators of the active sJIA [25, 32, 33]. Taken together, our findings implied that the expression of IL-37 is positively correlated with the disease activity of sJIA and may be a potential biomarker of active disease.

Previous studies have demonstrated that pro-inflammatory cytokines IL-1β, IL-6, TNF-α and IFN-γ markedly increased in sJIA patients and were closely related to the inflammation of sJIA [3, 28]. Clinical trials showed that antibodies of these pro-inflammatory cytokines could effectively alleviate the disease severity in patients with sJIA [7, 34–36]. Therefore, blocking these cytokines expression is a promising strategy for the development of novel anti-sJIA therapies. In accordance with previous data, we observed the higher plasma levels of these cytokines as well as IL-37 in sJIA patients than in HCs. We also found that the plasma levels of IL-37 were positively correlated with major pro-inflammatory cytokines TNF-α and IL-6 in patients with sJIA. Previous studies reported that IL-37 could be effectively induced synthesis by TNF-α in intestinal epithelial cells and PBMCs from healthy controls [37]. In addition, TNF-α also promoted the production of pro-inflammatory cytokines, such as IL-6, which in turn synergized with TNF-α to boost the inflammatory response [38]. Together with these data, we supposed that the uncontrolled inflammation might be due to the inadequate anti-inflammatory cytokines to completely neutralize excessive pro-inflammatory cytokines in the development of sJIA.

Recent studies have revealed that sJIA pathogenesis likely follows a biphasic course [39]. Pro-inflammatory cytokines driving the systemic disease by an innate immune response is the initial phase. Some inflammatory mediators dominating chronic arthritis by adaptive immune response is the second phase. Interestingly, our Spearman’s correlation analysis showed that plasma IL-37 levels were positively correlated with the inflammatory cytokine IL-17, and negatively correlated with the inflammatory mediator GM-CSF. IL-17 and GM-CSF are signature cytokines of Th17 cells, which have demonstrated major enrichment in the inflamed joints of children with JIA and correlated with the severity of disease [39, 40]. IL-17 drives chronic arthritis by triggering expressions of cytokines IL-1β and IL-6 and contributes to the balance of Th1/Th2 cells [41]. Kessel et al. found that pro-inflammatory cytokine environments can drive IL-17 overexpression by γ/δ T Cells in sJIA [39, 40]. Especially, Liang Ye et al. have demonstrated IL-37 inhibits IL-17 and IL-17—triggering cytokines production in RA patients [29]. GM-CSF is a potent inflammatory mediator that is responsible for recruitment and activation of immune cells. GM-CSF combined with Th2-secreted cytokine IL-4 can inhibit constitutive IL-37 expression [18]. Thus, it is reasonable to observe the negative correlation between plasma IL-37 levels and GM-CSF levels in sJIA patients. Recent study has reported that GM-CSF–expressing T cells were enriched in the joints of sJIA patients, highlighting the potential role of Th17-related cytokines in the pathology of sJIA [40]. As mentioned above, we speculate that IL-37 might not only participate in limiting excessive inflammation in innate immune response but also involve in chronic anti-inflammatory process by adaptive immunity in the development of sJIA.

The cumulative evidence suggested that IL-37 can suppress inflammatory response, possibly by reducing the production of pro-inflammatory cytokines induced by Toll-like receptor (TLR) agonists in several autoimmune and inflammatory diseases [18, 26]. In vivo experiments found that silencing endogenous IL-37 with small interfering RNAs in PBMCs dramatically upregulated the secretions of IL-1β, IL-6, and TNF-α after LPS treatment [18]. Additionally, in vitro experiments revealed that recombinant IL-37 proteins effectively inhibit the production of IL-1β, IL-6, and TNF-α in PBMCs from SLE, AS and AOSD patients [12, 13, 16]. An interesting question that emerges from these findings is “What is the function of IL-37 expression in the process of sJIA inflammatory response?” To answer this question, the rhIL-37 protein was used to stimulate PBMCs from patients with sJIA and healthy controls. Our study is the first to show that rhIL-37 could downregulate expressions of the critical pro-inflammatory cytokines TNF-α, IL-6, and IL-17 after LPS stimulation in PBMCs of sJIA patients, which have no effect on the productions of the pro-inflammatory cytokines in PBMCs of healthy controls. It was shown recently that pro-inflammatory cytokines TNF-α, IL-6, and IL-17 can induce the high expression of IL-37 [18]. Based on these information, it is plausible to suggest that the increased IL-37 production induced by the high expressions of pro-inflammatory cytokines in sJIA could in turn repress their excessive production through a feedback regulatory loop only in the inflammatory phase.

## Conclusions

Altogether, we demonstrated that the IL-37 levels were elevated in active sJIA patients, and were correlated with the sJIA disease activity and clinical features. More importantly, our study showed that IL-37 level were associated with inflammatory cytokines in innate as well as adaptive immune response. In vitro experiments revealed that IL-37 may mediate its anti-arthritic effects through inhibiting the production of pro-inflammatory cytokines. These data implicated that IL-37 probably played an important role in the inhibition of pathogenesis of sJIA, and suggested a possible new therapeutic target for sJIA. Further research is necessary to determine the source of IL-37 expression, the regulatory mechanisms and signal pathways of IL-37 in the process of sJIA inflammatory response.

## Additional file

**Additional file 1: Fig S1.** Comparison of plasma cytokines between sJIA patients and HCs. Plasma IL-1β (**a**), IL-6 (**b**), TNF-α (**c**), IL-17 (**d**), IFN-γ (**e**) and GM-CSF (**f**) protein levels among patients from sJIA, active sJIA (n = 23), inactive sJIA (n = 23) as well as HCs (n = 30) were determined by ELISA. Each symbol represents an individual patient with sJIA and HCs. Horizontal lines indicate median values. The data represent the mean ± SD. ***P < 0.001, **P < 0.005, *P < 0.05 by Student’s *t* test.

## Abbreviations

IL-37: interleukin-37
PBMCs: peripheral blood mononuclear cells
sJIA: systemic juvenile idiopathic arthritis
HCs: healthy controls
RT-PCR: real-time polymerase chain reaction
ELISA: enzyme-linked immunosorbent assay
TNF-α: tumor necrosis factor-α
IL-1β: interleukin-1β
IL-6: interleukin-6
IL-8: interleukin-8
IL-17: interleukin-17
IFN-γ: interferon-γ
AS: ankylosing spondylitis
SLE: systemic lupus erythematosus
RA: rheumatoid arthritis
AOSD: adult-onset Still’s disease
JADAS-27: Juvenile Arthritis Disease Activity Score in 27 joints
LPS: lipopolysaccharide
LB: lysogeny broth
EDTA: ethylene diamine tetraacetic acid
rhIL-37: recombinant human IL-37
ESR: erythrocyte sedimentation rate
CRP: C-reactive protein
HGB: hemoglobin
PLT: platelet
AST: aspartate transaminase
ALT: alanine transaminase
SF: synovial fluid
TLR: Toll-like receptor
