# Supplementary figures and images for: Plasma interleukin-37 is increased and inhibits the production of inflammatory cytokines in peripheral blood mononuclear cells in systemic juvenile idiopathic arthritis patients
Source: J Transl Med. 2018 Oct 11;16:277. doi: 10.1186/s12967-018-1655-8 (PMC6180625; doi:10.1186/s12967-018-1655-8)

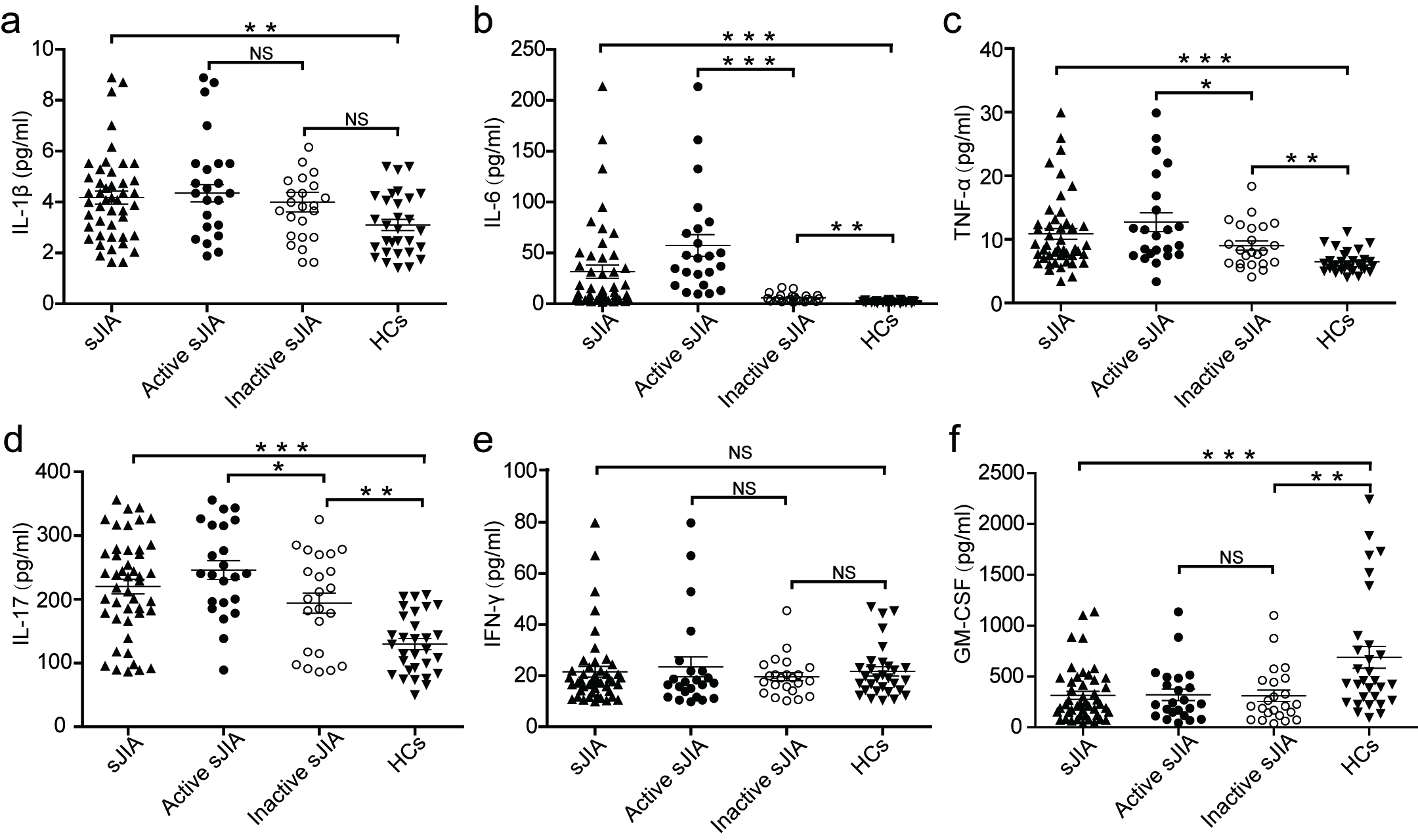

Supplement: Supplementary file 1 — Additional file 1: Fig S1. Comparison of plasma cytokines between sJIA patients and HCs. Plasma IL-1β (a), IL-6 (b), TNF-α (c), IL-17 (d), IFN-γ (e) and GM-CSF (f) protein levels among patients from sJIA, active sJIA (n = 23), inactive sJIA (n = 23) as well as HCs (n = 30) were determined by ELISA. Each symbol represents an individual patient with sJIA and HCs. Horizontal lines indicate median values. The data represent the mean ± SD. ***P < 0.001, **P < 0.005, *P < 0.05 by Student’s t test. [file 12967_2018_1655_MOESM1_ESM.tif]
